# A cerebellar mechanism for learning prior distributions of time intervals

**DOI:** 10.1038/s41467-017-02516-x

**Published:** 2018-02-01

**Authors:** Devika Narain, Evan D. Remington, Chris I. De Zeeuw, Mehrdad Jazayeri

**Affiliations:** 1 Department of Brain and Cognitive Sciences, Massachusetts Institute of Technology, Cambridge, MA 02139 USA; 2 McGovern Institute for Brain Research, Massachusetts Institute of Technology, Cambridge, MA 02139 USA; 3 Department of Neuroscience, Erasmus Medical University, 3015 CN Rotterdam, The Netherlands; 4 Netherlands Institute of Neuroscience, 1105 BA Amsterdam, The Netherlands

## Abstract

Knowledge about the statistical regularities of the world is essential for cognitive and sensorimotor function. In the domain of timing, prior statistics are crucial for optimal prediction, adaptation and planning. Where and how the nervous system encodes temporal statistics is, however, not known. Based on physiological and anatomical evidence for cerebellar learning, we develop a computational model that demonstrates how the cerebellum could learn prior distributions of time intervals and support Bayesian temporal estimation. The model shows that salient features observed in human Bayesian time interval estimates can be readily captured by learning in the cerebellar cortex and circuit level computations in the cerebellar deep nuclei. We test human behavior in two cerebellar timing tasks and find prior-dependent biases in timing that are consistent with the predictions of the cerebellar model.

## Abstract (web summary)

Human timing behavior is biased towards previously encountered intervals and is predicted by Bayesian models. Here, the authors develop a computational model based in properties of the cerebellum to show how we might encode time estimates based on prior experience.

## Introduction

Human timing behavior is associated with two robust properties. First, response times are biased toward the mean of previously encountered intervals^1^ and second, the bias is stronger for longer sample intervals^2,3^. Bayesian models predict both properties accurately^2–5^. They predict biases that reflect the use of prior knowledge and predict larger biases for longer intervals, which are more uncertain due to scalar variability in timing^6,7^. Despite the remarkable success of Bayesian models in describing human timing behavior, little is known about the brain structures and mechanisms that support learning of prior distributions to enable Bayesian inference.

Several considerations suggest that the cerebellum might play a role in learning sub-second to second temporal associations in sensorimotor behavior^8,9^. First, the rapid learning in behavioral timing experiments^2,10,11^ is consistent with the relatively fast learning dynamics in the cerebellum^12^. Second, lesions of the cerebellum impact temporal coordination without necessarily influencing movement ability^13^. Third, human neuroimaging experiments implicate the cerebellum in timing^14,15^. Fourth, work in non-human primates suggests that the cerebellum is involved in a range of sensorimotor and non-motor timing tasks, from temporal anticipation during smooth pursuit^16,17^ to detecting oddballs in rhythmic stimuli^18^, to timing self-initiated movements^19^. Finally, studies of eyeblink conditioning in humans, as well as numerous animal models^20,21^ suggest that the cerebellum is one of the key nodes involved in learning the interval between conditioned and unconditioned stimuli.

Cerebellar circuits can learn multiple time intervals simultaneously. For example, in eyeblink conditioning, animals can concurrently acquire differently timed conditioned eyelid responses associated with distinctive conditioned stimuli (CS)^22^. The ability to acquire more than one interval suggests that the cerebellum might have the capacity to learn a range of previously encountered intervals. This intriguing possibility suggests that the cerebellum might play a functional role in Bayesian computations that rely on knowledge about the prior probability distribution of time intervals. Here we propose a theoretical model called TRACE (temporally reinforced acquisition of cerebellar engram) that synthesizes known anatomical and physiological mechanisms of the cerebellum to explore how prior distributions could be encoded to produce Bayesian estimates of time intervals.

## Results

### Behavioral paradigm

**Fig. 1:**
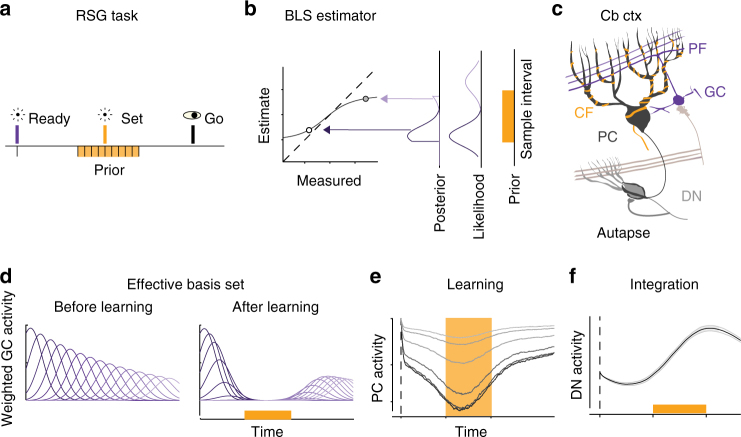
Task, cerebellar anatomy and the TRACE model. **a** Ready-Set-Go task. On each trial, participants measure a sample interval demarcated by two visual flashes– Ready (purple bar) and Set (orange bar) – and aim to reproduce that interval  immediately after Set (Go). The sample interval is drawn from a uniform prior distribution (orange distribution). **b** The Bayes-Least-Squares (BLS) estimator. A Bayesian observer computes the posterior based on the product of the prior and the likelihood function, and uses the mean of the posterior to estimate the interval. The plot shows the behavior of BLS for two measurements. The light and dark bell-shaped curves represent the likelihood function associated with those measurements. The BLS estimate is the expected value of the posterior, which is biased toward the mean of the prior (arrows) and away from the unity line (dashed). Due to scalar variability, the likelihood is wider for longer measurements (lighter likelihood function), resulting in a larger bias. **c** Schematic drawing of the relevant part of the cerebellar circuit. In the cerebellar cortex (Cb ctx), a Purkinje cell (PC, black) receives inputs from granule cells (GC, purple) via parallel fibers (PF), and from climbing fibers (CF, orange). PCs, in turn, project to and inhibit neurons in the dentate nucleus (DN), which additionally receive extra-cerebellar input (light brown) and autaptic (synapse onto self) input. **d** The effective basis set in TRACE, which reflects the GC basis set scaled by the GC to PC synaptic weights (i.e., Weighted GC activity). The plot shows weighted GC activity for a subset of GCs in the model. Left: Weighted GC activity before learning (sub-sampled). Right: Weighted GC activity after learning the prior. **e** PC activity profile based on TRACE simulations at different stages of  learning. The PC activity is shown after 10, 50, 100, 150, and 200 trials during learning (light to dark). **f** DN activity. The activity profile of DN reflects the integrated output of PCs, and is plotted after learning (trial 200). Shaded gray region indicates standard deviation. Traces in **e** and **f** do not correspond to any specific sample interval; instead, they show the expected response profiles in the absence of CF input (i.e., as if Set were never presented). Dashed lines in **e** and **f** correspond to the time of Ready, and the orange regions highlight the domain of the prior

To assess the potential role of the cerebellum in Bayesian time estimation, we focused on a simple time interval reproduction task (Fig. 1a). In this task, which we refer to as Ready-Set-Go (RSG), two cues, Ready and Set, demarcate a sample interval drawn from a prior distribution that participants estimate and subsequently reproduce. Previous work has shown that both humans^3–5^ and monkeys^23^ exhibit two classic features of Bayesian timing while performing this task (Fig. 1b): produced intervals are biased toward the mean of the prior distribution, and this bias is larger for longer and more uncertain intervals. We use this task to examine whether and how the cerebellum could acquire prior distributions of time intervals and compute Bayesian estimates of the measured intervals.

### TRACE model

The TRACE model consists of three components that are motivated by the known anatomy and physiology of the cerebellum (Fig. 1c). The first of these is a basis set, which represents reproducible and heterogenous patterns of activity across granule cells (GCs, Fig. 1d). The second is learning, which relies on known plasticity mechanisms at the synaptic junction between granule cell (GC) axons (parallel fibers, PF) and Purkinje cells (PCs, Fig. 1e). The third is integration, which captures transformations downstream of the cerebellar cortex (Fig. 1f).

### Basis set

The first component of TRACE is a heterogeneous temporal basis set across GC neurons (Fig. 1d). Recent experimental findings are consistent with the existence of such heterogeneity^24,25^ and models of cerebellar tasks have highlighted the computational advantage that such a basis set could confer upon learning temporal contingencies^26–28^. The mechanisms for the generation of such basis sets is not known but is likely to depend on sustained mossy fiber input. In certain behavioral paradigms, such as delay eyeblink conditioning, a continuous sensory input could provide the necessary extra-cerebellar drive. In other paradigms in which continuous sensory input is not present, such as trace eyeblink conditioning and the RSG task, this drive could be supplied by forebrain areas capable of generating self-sustained activity^29^.

We assumed that the Ready cue triggers a reproducible basis set across GCs. We modeled this basis set as Gaussian kernels across time (Supplementary Fig. 1a). A large body of literature in animal models and humans suggests that the representation of time in the brain is subject to scalar noise; i.e., noise whose standard deviation scales with elapsed time^6^. We used a probabilistic model to characterize the effect of scalar noise on the expected profile of the basis set (Supplementary Fig. 1b). The model demonstrated that scalar noise distorts the expected profile of the kernels in two ways: it causes amplitudes to decay and widths to increase with time. These effects were accurately captured by augmenting the basis set activity profiles (*r*(*t*)) with an exponential decay in amplitude *g*(*t*) and a linear increase in width (*σ*_basis_) with time (Eq. 1, Fig. 1d, Supplementary Fig. 1b; see Methods section). Furthermore, we verified that both properties of decay and increased width in a basis set might emerge naturally as a consequence of accummulating noise through recurrent interactions in a network of neurons (Supplementary Fig. 2).

1 $$r_i\left( t \right) = g\left( t \right){\textstyle{1 \over {\sqrt {2\pi \sigma _{{\rm basis}_i}^2} }}}{\rm e}^{\frac{{ - \left( {t - t_i} \right)^2}}{{2\sigma _{{\rm basis}_i}^2}}}$$

where *t*_i_ corresponds to the time after *Ready* when the *i*-th GC activity reaches its peak, and $g\left( t \right) = {\rm e}^{\frac{{ - t}}{{\tau _{{\rm basis}_i}}}}$.

### Learning

The acquisition of time intervals in the cerebellar cortex for eyeblink conditioning is primarily attributed to synaptic mechanisms that can suppress the activity of PCs^30^. This decrease in activity is engendered or extinguished by a myriad pre- and postsynaptic plasticity mechanisms acting among PCs and interneurons^31^. However, most of these are synergistically and bidirectionally regulated by climbing fiber (CF, Fig. 1c) activity^32^. For the TRACE model, we summarize the overall synaptic contributions of these mechanisms into common learning and restoring mechanisms, which we broadly refer to as long-term depression and potentiation (LTD and LTP)^33–35^. Indeed, depression of parallel fiber synaptic weights onto Purkinje cells through LTD generally depends on the conjunctive activation of GCs and complex spikes triggered by CFs, whereas LTP is believed to occur when GCs are active in the absence CF activity. Learning in TRACE therefore depends on these two complementary mechanisms. We assumed that Set activates CFs and causes LTD in the subset of synapses that are activated by GCs shortly before the time of Set. Because the activity of GCs is triggered by Ready, this plasticity mechanism causes an interval-dependent suppression in PC activity that reflects the Ready-Set interval. The LTP, on the other hand, potentiates those GC to PC synapses associated with GCs that are active in the absence CF activity. The learning rule we used (Eq. 2) is similar to previous modeling work on LTP and LTD learning in the cerebellum^36^. We formulated it as:

2 $$\frac{{{\rm d}w_i}}{{{\rm d}t}} = - \frac{1}{{\tau _{{\rm ltd}}}}r_i\left( {t_{\rm s} - \varepsilon } \right)\delta(t-t_{\rm s}) + \frac{1}{{\tau _{{\rm ltp}}}}\left( {w_{\rm o} - w_i} \right)$$

LTD (first term) is activity-dependent (dependent on *r*_*i*_(*t*)), and effective only for eligible synapses. A synapse was considered eligible if the corresponding GC was active within an eligibility trace (*ε*) before CF activation. Based on previous reports for eyeblink conditioning^27,37^, we estimated *ε* to be 50 ms. The conjunction of GC and CF activity is represented by a delta function that was nonzero only at the time of *Set* (*δ*(*t-t*_s_)). In contrast, LTP (second term) acted as a restoring force driving the synaptic weight towards a baseline (*w*_o_) when GCs fired in the absence of CF stimulation. This learning rule was governed by two free parameters: the time constants of LTP and LTD (*τ*_ltd_ and *τ*_ltp_).

The LTD component of this learning rule permits each presented sample interval to reduce the synaptic weight of the subset of GCs in the basis set that are eligible at the time of Set. Consequently, multiple exposures to sample intervals drawn from a prior distribution (Fig. 1a, orange) allow GC–PC synapses to gradually acquire a representation of the full prior distribution (Fig. 1d, right). LTP, on the other hand, gradually washes out LTD, thereby allowing adaptation. More specifically, LTP allows synaptic weights to have a stronger footprint for intervals that are presented more frequently. LTP also allows synapses to represent the most recently encountered time intervals. The success of the model and the speed with which the model adapts to changes depends on the relative time constants associated with LTP and LTD. The behavior of TRACE is relatively robust to variations of these time constants  as long as LTD is stronger than LTP (Supplementary Fig. 3).

Modification of GC–PC synapses directly impacts PC activity, since it represents the net granule cell activity filtered by its synaptic weight. We modeled PC activity (*V*_pc_, Eq. 3) as the linear sum of GC basis set activity filtered by the GC–PC synaptic weights (*w*_i_). Accordingly, PC activity is influenced by both the response profile of the GC basis set and the learned synaptic weights (Fig. 1e). This enables PCs to encode a composite variable that carries information about both uncertainty in the measurement (via the basis set) and the prior distribution (via the synaptic weights).

3 $$V_{{\rm pc}}\left( t \right) = {\sum} {w_ir_i\left( t \right)}$$

An implicit assumption of this learning scheme is that the circuit must correctly route stimuli such that Ready would activate GCs and Set would activate CFs; however, no such precise routing is necessary. Since Ready and Set are provided by similar visual inputs, it is conceivable that they activate both pathways and may potentially interfere with the behavior of TRACE in two ways. The first concern is the hinderance of learning if the CF is triggered by Ready, for which, there ought to be another preceding stimulus that would activate a reproducible basis set. Since no such stimulus is present in the RSG task, the activation of CF by Ready has no effect on the behavior of TRACE. The second potential concern is the reactivation of the basis set by the Set cue. Because GC to PC synapses undergo LTP in the absence of CF activity, this reactivation could lead to stronger LTP and shorter timescales of learning. We investigated this possibility quantitatively and found that TRACE’s steady-state behavior is robust as long as LTD is stronger than the effective LTP (Supplementary Fig. 3), an assumption consistent with previous work on cerebellar learning^38^.

### Integration

The last stage of TRACE is concerned with the transformation of PC activity in the cerebellar deep nuclei (Fig. 1f). We focused our attention on the caudal region of the dentate nucleus where timing signals in both motor^19^ and non-motor timing tasks^18^ have been observed at the level of individual dentate neurons (DNs). This region contains large columnar neurons with three major synaptic inputs: extra-cerebellar currents, inhibition from PCs, and autaptic currents (neuron’s axon collaterals synapsing back on itself as illustrated in Fig. 1c)^39^. Accordingly, the membrane potential of DNs can be modeled as follows:

4 $$\frac{{{\rm d}V_{{\rm dn}}\left( t \right)}}{{{\rm d}t}} = - V_{{\rm dn}}\left( t \right) + g_{{\rm dn}}V_{{\rm dn}}\left( t \right) - g_{{\rm pc}}V_{{\rm pc}}\left( t \right) + I_{{\rm eff}}$$

where *g*_dn_, *g*_pc_, and *I*_eff_ correspond to the conductance associated with the autaptic input, the conductance associated with PCs, and the remaining effective input driving DNs, respectively.

In this model, the transformation of *V*_pc_(*t*) by the DNs depends on two main factors, the autaptic conductance (*g*_dn_), and the effective input drive (*I*_eff_). Similar to previous work on autaptic and recurrent architectures^40,41^, *g*_dn_ currents counteract the leakage current and allow the neuron to act as an integrator. For the remainder of the manuscript, we assume that *I*_eff_, which is a constant positive drive, is equal to the average of *g*_pc_*V*_pc_(*t*) over time. We later show that relaxation of this assumption does not impact the model behavior (Supplementary Fig. 4a–c). We also assume that DNs act as perfect integrators (i.e., *g*_dn_ = 1) and integrate the inputs from PCs and the input current (*I*_eff_ − *g*_pc_*V*_pc_(*t*)). We also show that the model is robust to changes in the value of *g*_dn_ (Supplementary Fig. 4d–f). With these assumptions, the membrane potential of the DN (*V*_dn_, Eq. 5) can be computed as follows:

5 $$V_{{\rm dn}}(t) = {\int} {\left( {I_{{\rm eff}} - g_{{\rm pc}}V_{{\rm pc}}\left( t \right)} \right){\rm d}t}$$

### Bayesian estimation by TRACE

**Fig. 2:**
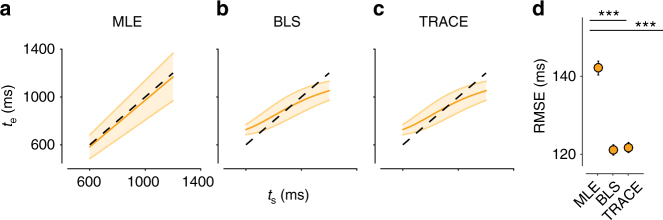
Bayesian estimation by TRACE. **a** The maximum likelihood estimator (MLE). The plot shows the mean (orange line) and standard deviation (shaded area) of interval estimates (*t*_e_) derived from MLE as a function of the sample interval (*t*_s_) in the RSG task. MLE provides  accurate estimates on average (orange line close to the unity dashed line), but the standard deviation of *t*_e_ is relatively large and increases with *t*_s_. **b** The Bayes-Least-Squares (BLS) estimator. BLS estimates are biased away from *t*_s_ and toward the mean of the prior but are substantially less variable (smaller shaded region). **c** The output of TRACE. TRACE behaves in a manner similar to BLS. **d** Root-mean-square error (RMSE) between *t*_e_ and *t*_s_ for MLE, BLS, and TRACE. Both BLS and TRACE outperformed MLE (paired *t*-test BLS-MLE RMSE: *t*_1998_ = 739, *p* $\ll$ 0.001 TRACE-MLE: *t*_1998_ = 731, *p* $\ll$ 0.001). Asterisks indicate *p* $\ll$ 0.001. Error bars indicate standard deviation across runs of RMSE calculated over all sample intervals

We compared the behavior of TRACE to the Bayes-Least-Squares (BLS) estimator, which establishes a nonlinear function that transforms measured variables to optimal estimates. This transformation minimizes root-mean-square error (RMSE) by biasing estimates toward the mean of the prior, which leads to a substantial reduction in variability. In the RSG task, due to scalar variability of measurements, the magnitude of the bias is larger for longer intervals. The integrated output of TRACE (i.e., the DN activity) accurately captured these characteristics, and similar to BLS, outperformed a suboptimal maximum likelihood estimator (Fig. 2). The similarity between TRACE and BLS is remarkable given that TRACE is a physiologically inspired model whereas BLS represents optimal performance by an ideal observer (Figs. 2b-d). Therefore, TRACE captured both temporal uncertainty and prior-dependent biases in a manner highly consistent with Bayesian estimation theory. Finally, this behavior of TRACE was robust to variation of parameters in the basis set, learning and integration (Supplementary Figs. 3–5).

**Fig. 3:**
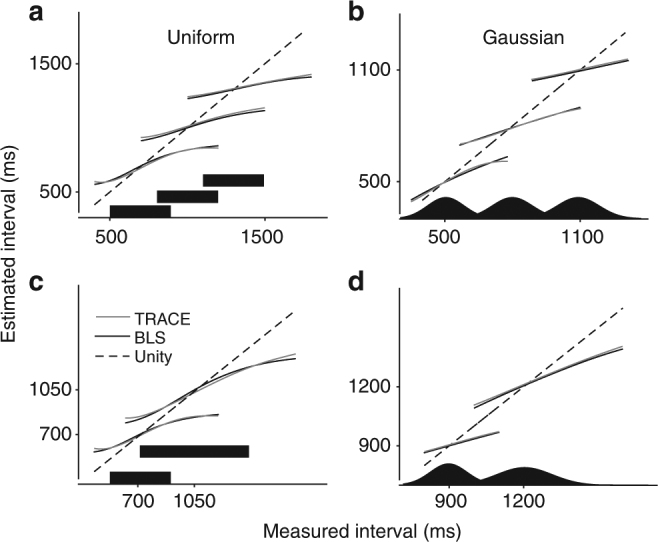
Comparison of TRACE and BLS for different priors. **a** Estimates derived from Bayes-Least-Squares (BLS) (black line) and TRACE (gray line) for three different uniform prior distributions (solid black) with different means. **b** Same as **a** for three Gaussian prior distributions. **c** Same as **a** for two uniform priors with different means and widths. **d** Same as **c** for Gaussian priors. We used a linear scaling parameter to calibrate the output of TRACE and added the mean of the prior as an offset so that TRACE and BLS could be compared directly

**Fig. 4:**
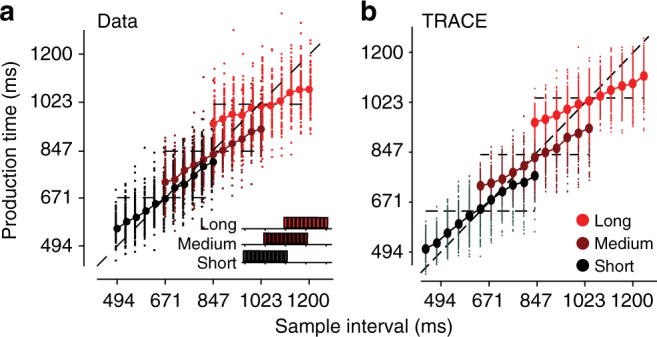
Comparison of TRACE with human behavior in the RSG task. **a** Human behavior during the RSG task with three different prior distributions, Short (black), Medium (dark red) and Long (red). Data adapted from previous work ^3^. Note that bias toward the mean of the prior is larger for longer interval ranges. **b** The corresponding prediction from TRACE. Dots and circles mark individual responses and averages respectively. To directly compare TRACE and human behavior, scalar noise was added to the output of TRACE to simulate responses (Methods section). Parameters of the model were not tuned to the data and a single-linear scaling factor was used to calibrate model output for all conditions^3^

Next, we tested whether TRACE could perform Bayesian estimation in contexts where the prior distributions were uniform or Gaussian with different means and standard deviations (Fig. 3). The output of TRACE matched the behavior of a BLS estimator across all conditions without any need to adjust model parameters. In the case of uniform priors with different means, we additionally compared the behavior of TRACE to that of human subjects reported previously^3^ and found that the model was able to accurately capture the observed biases (Fig. 4), especially the tendency for increased biases for prior distributions over longer intervals (compare slopes of red and black responses in Fig. 4a, b).

### TRACE learning dynamics

The learning dynamics in TRACE can be evaluated in terms of two variables, the asymptotic change in synaptic weights and the effective time constant at which synapses reach that asymptotic value. Both the asymptotic weight change and the effective time constant are influenced by the time constants associated with LTP to LTD. Overall, *τ*_ltp_ must exceed *τ*_ltd_ for stable learning to occur. As *τ*_ltp_ increases, the model establishes a stronger and more resilient footprint of previously encountered time intervals (larger weight change). This however, comes at the cost of slower restoration (or forgetting). This results in slower adaptation to recent changes.

**Fig. 5:**
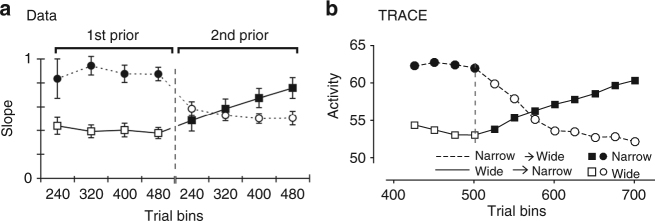
Comparison of TRACE with human behavior during learning. **a** Human behavior during transitions between a wide and narrow prior distribution in a time interval reproduction task similar to RSG. The data are adapted from previous work^2^. The results were quantified by a previously reported ‘slope’ parameter that quantified the strength of the bias towards the mean of the prior (in this case, higher slope represents more bias toward the prior). The switch (vertical dashed line) from narrow (filled symbols) to wide (open symbols) decreases the influence of the prior and vice versa. **b** The corresponding prediction from TRACE (Methods section) indicates the expected timescales during the course of learning such priors. The change in PC activity compared to baseline is plotted on the ordinate (in arbitrary units). TRACE parameters were the same as those used in RSG and were not fit to the data

We tested the model’s ability to capture the dynamics of learning prior distributions in a previous behavioral study involving a time reproduction task similar to RSG^2^. In this study, participants learned a prior distribution when, unbeknownst to them, the prior distribution was altered. Interestingly, participants’ adjustments were slower when the prior switched from a wide to narrow distribution of intervals compared to vice versa (Fig. 5a). Similar results for switching between wide and narrow priors have been reported in other Bayesian tasks in the sensorimotor domain^42,43^. TRACE exhibited the same behavior as evidenced in the changes of weights in the GC–PC synapses: learning was relatively slower after switching from a wide to a narrow prior (Fig. 5b). This behavior can be understood by the conjoint operations of LTP and LTD in response to the two kinds of switches. When the prior switches from wide to narrow, LTP restores the depression associated with intervals that are no longer presented. In contrast, when the prior switches from narrow to wide, LTD creates a footprint for the newly presented intervals. Since LTP is slower than LTD (to help retain information from past trials more effectively), learning in the former conditions proceeds more slowly.

### Comparing TRACE output to neural activity

**Fig. 6:**
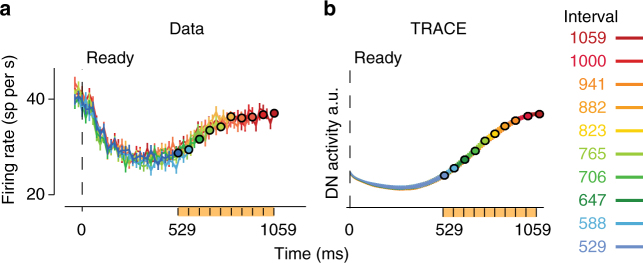
Comparison of TRACE with electrophysiology data. **a** Physiology. Activity in the lateral intraparietal (LIP) area of monkeys performing the RSG task is modulated nonlinearly during the range of the prior (orange) between 529 and 1059 ms. The data adapted from previous work^23^. **b** The corresponding prediction from TRACE. The output of TRACE is shown with arbitrary units. Error bars indicate SEM. Note that the early modulation of LIP activity near the time of *Ready*, which is absent in TRACE, is due to the presentation of a visual stimulus in the response field of LIP neurons before the presentation of *Ready*. The relevant region of comparison between the two panels is within the domain of the prior distribution

In TRACE, DN neurons integrate the activity of PCs across time. This makes a specific prediction for how the output of the cerebellum (i.e., DN activity) would reflect previously encountered time intervals (i.e., prior distribution) in RSG. A recent study characterized activity in the lateral intraparietal cortex (LIP) of monkeys in the RSG task with a uniform prior ranging between 529 and 1059 ms^23^. Since previous work has shown that LIP neurons receive transthalamic input from DNs^44,45^, we compared the output of TRACE with the LIP activity. LIP neurons were modulated throughout the Ready-Set epoch and displayed a nonlinear response profile during the range of the prior distribution (Fig. 6a; between 529 and 1059 ms). This profile was highly similar to the output of TRACE (Fig. 6b). This similarity is consistent with TRACE being involved in Bayesian inference of time intervals. TRACE thus makes the prediction that the nonlinear response profiles observed in LIP during prior-dependent timing tasks may be inherited from the output of the cerebellum. A salient difference between LIP and TRACE is that LIP responses were strongly modulated near the time of Ready. This modulation, however, is due to the onset of a saccadic visual target before Ready^23^. As expected, this visually-triggered response is absent in TRACE.

### Model lesions

**Fig. 7:**
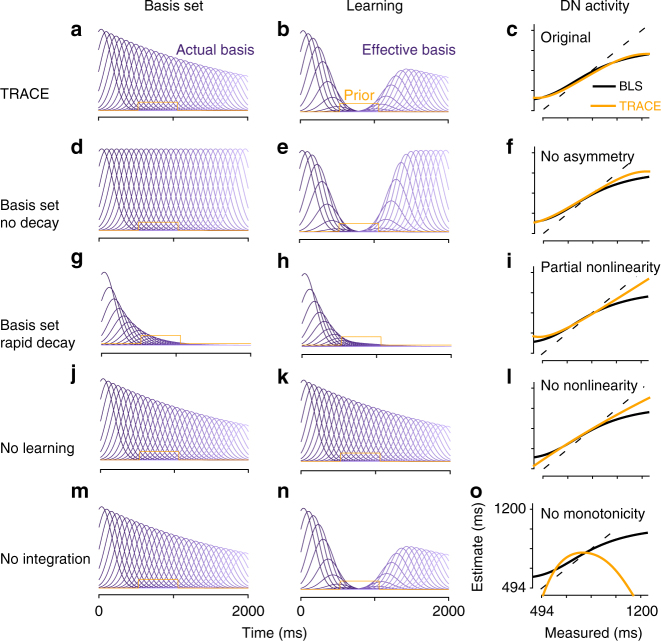
TRACE in-silico lesions. **a-c** TRACE with all three model components intact. **a** The basis set. **b** The basis set weighted by modified GC to PC synapses after learning of the prior distribution has reached steady state. **c** The linearly transformed output of TRACE (orange) superimposed on the BLS function (black). **d**–**f** Same format as for **a**–**c** when there is no decay in the basis set. The asymmetry in TRACE output is lost. **g**–**i** Same as earlier panels with rapid decay of basis set. The nonlinear bias for longer intervals is abolished. **j**–**l** If no learning takes place, then there is no nonlinearity in the model output. **m**–**o** If no integration takes place, there is no monotonically increasing output, leading to significant deterioration in performance

To evaluate the three key components of TRACE (basis set, learning, and integration), we examined its behavior after ‘lesioning’ each of these elements (Fig. 7). For the first component, we first simulated a variant of TRACE in which the basis set did not decay with time (Fig. 7d-f). This disruption led to a loss of the interval-dependent asymmetry in the bias (i.e., bias for longer intervals was not stronger than that for shorter intervals), which is a key feature of Bayesian inference in timing. In contrast, if the basis set decays too rapidly, the model fails to capture prior-dependent biases for longer intervals (Fig. 7g-i). From an experimental point of view, these results indicate a direct relationship between behavioral output and the temporal jitter (i.e., noise) in the basis set, and could be verified by injecting suitable forms of noise within the GC population. The learning element of the model also proved to be crucial. When no learning was permitted, TRACE was insensitive to the prior distribution (Fig. 7j-l). In other words the model predicts that if LTD pathways were knocked-out, interval learning and therefore any prior-dependent representations would be disrupted. Finally, without the integration component, the time course of the model output was unable to reflect the monotonic increase in time estimates with duration (Fig. 7m-o). This component of the model could be verified by first characterizing and then perturbing the hypothesized circuitry in the deep nuclei responsible for integration. These results validate the necessity of  all three components of the model in inducing  Bayesian behavior.

### Bayesian biases in cerebellar timing tasks

To establish a stronger link between TRACE and cerebellar timing tasks, we investigated whether biases toward the statistics of prior distributions could be demonstrated in two cerebellar timing tasks: (1) trace eyeblink conditioning using relatively short trace intervals that minimize forebrain involvement in humans^46,47^, and (2) synchronization-continuation, which is thought to depend on the cerebellum^9,48^.

**Fig. 8:**
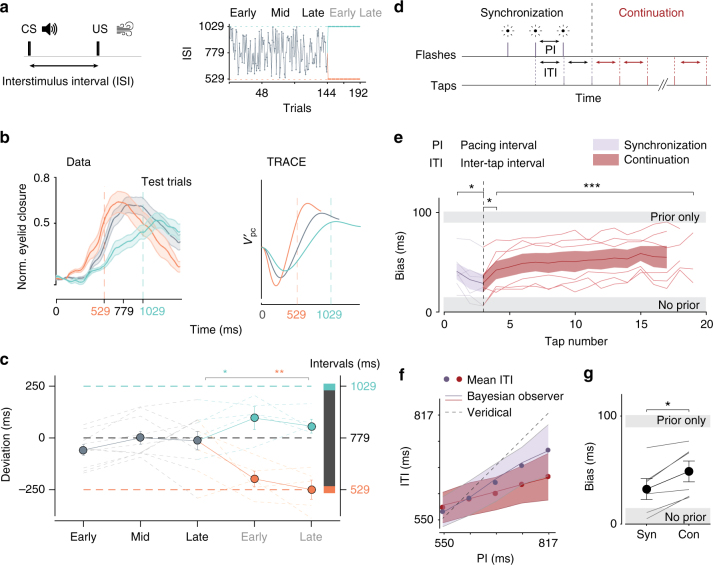
Prior-dependent biases in cerebellar timing tasks in humans. **a** Left: trace eyeblink conditioning using tone as the conditioned stimulus (CS) and airpuff as unconditioned stimulus (US), with interspersed test trials (CS-only). The two stimuli were separated by an interstimulus interval (ISI). Right: ISI within a behavioral session was drawn randomly from a uniform distribution ranging between 529 and 1059 ms (gray) for the first 144 trials. Afterwards, ISI was switched to a single valued distribution (i.e., delta function), either a fixed 529 ms (orange) or a fixed 1059 ms (green). **b** Left: normalized average eyeblink closure traces as a function of time after CS in the test trials before (gray) and after (orange/green) the switch. Right: TRACE predictions for the same distributions. **c** Deviation of peak eyelid closure time from the mean of the wide uniform ISI distribution. The deviation is computed in early (1–48), middle (49–96), and late (97–144) stages before the switch, and in early (145–168) and late (169–192) stages after the switch. Circles indicate averages and faint lines individual participants. Error bars indicate SEM. **d** Synchronization-Continuation task. In the ‘Synchronization’ phase, participants tapped in synchrony with isochronous flashes (with pacing interval PI) and continued tapping in the ‘Continuation’ phase with an inter-tap-interval (ITI). PI was a fixed within trials and sampled from a uniform distribution across trials. **e** Bias in the synchronization (purple) and continuation (red) phases for all participants (thin lines) and their averages (thick lines). The magnitude of bias for all participants lay between two extremes, a hypothetical observer that does not rely on the prior (lower gray bar, no prior), and a hypothetical observer that only uses the mean of the prior (upper gray bar, prior only). **f** An example participant. Filled circles represent the mean ITI corresponding to each PI for synchronization (purple) and continuation (red). Solid lines correspond to a Bayesian observer model fit to the participant’s data. Dashed line indicates veridical performance. **g** Comparison of bias between synchronization and continuation phases for individual participants (gray) and averages (solid black). In **e**, **f** shaded regions and error bars show SD

In the trace conditioning experiment (Fig. 8a-c), participants (*N* = 10) received repeated pairings of a transient tone and an airpuff serving as the conditioned and unconditioned stimuli respectively (CS and US). We varied the inter-stimulus-interval (ISI) between CS and US while characterizing the kinematics of  predictive eyelid closure, which served as the conditioned response (CR). In the first 144 trials, the ISI was drawn from a wide uniform prior distribution ranging from 529 to 1059 ms (Fig. 8a). Thereafter, unbeknownst to participants, the ISI was switched to a narrow distribution, either a fixed interval of 529 (short) or 1059 ms (long). At regular but unpredictable intervals, we interspersed test trials in which the US was omitted and the CS was presented alone. These test trials were used to quantify the CR and assess learning.

During exposure to the wide prior, the peak of the CR gravitated toward the mean of the ISI distribution (Fig. 8b, c). Furthermore, when the ISI distribution was switched from wide to narrow, the peak of the CR reflected biases toward the new ISIs (Fig. 8b, repeated measures analysis of variance, main effect for switch to short *F*_short(2,8)_ = 13.6, *p* < 0.01 and long intervals, *F*_long(2,8)_ = 6.8, *p* = 0.02). Both the coincidence of the peak response with the mean of the wide prior before the switch and the shift of the peak time after the switch indicate that learning was sensitive to the temporal statistics of the ISI. The time course of the CR across the three prior conditions (Fig. 8b, left) revealed a systematic relationship between the amplitude and time of the peak eyelid closure: earlier CRs exhibited larger amplitudes. This relationship was accurately captured by TRACE (Fig. 8b, right) due to its decaying basis set, which results in stronger changes in disinhibited PC response profile for shorter intervals $V_{{\rm pc}}^\prime$.

In the synchronization-continuation task (Fig. 8d), participants (*N* = 6) were presented with the first four beats of an isochronous rhythm (synchronization phase) and were asked to tap a key in synchrony with the third and fourth beats and continue tapping afterwards at the same rate (continuation phase). On every trial, the pacing interval (PI) between the beats was drawn from a discrete uniform distribution, ranging between 550 and 817 ms. In our analysis, we considered the first tap during the continuation phase as a synchronization tap. As evident from the behavior of individual participants (Fig. 8e, f), inter-tap-intervals (ITIs) were biased toward the mean of the prior in both synchronization and continuation phases. Using a summary statistic, Bias, which measured the difference between average ITI and PI, we verified that ITIs were biased toward the mean of the prior in both phases (Fig. 8f, g) and that the magnitude of the bias was larger in the continuation phase (paired *t*-test *t*_5_ = 4; *p* = 0.01). This behavior is consistent with results from previous time interval reproduction tasks^2,3^ and is indicative that participants integrate prior knowledge with noisy sensory information in a Bayesian fashion. From a Bayesian perspective, this is not surprising. As the memory of the pacing interval degrades, participants should rely more on prior knowledge during the continuation phase^49^. A more detailed inspection of performance throughout trials was consistent with this interpretation. Following the final interval in the synchronization phase, Bias increased (paired *t*-test *t*_5_ = 3; *p* = 0.03) and then further increased throughout the continuation interval (RM ANOVA *F*_(13,65)_ = 5.1, *p* $\ll$ 0.001). Conversely, Bias decreased through the synchronization phase as additional measurements were provided by the pacing stimuli (RM ANOVA *F*_(2,10)_ = 6.1, *p* = 0.02).

## Discussion

Numerous behavioral studies have shown that humans rely on prior knowledge to mitigate the uncertainty in sensory measurements, as predicted by Bayesian theory^2,3^. This raises the possibility that brain circuits have the capacity to encode prior distributions and use them to optimize behavior. However, where and how prior distributions are represented in the brain is a matter of debate. In sensory domains where prior knowledge is characterized by the natural statistics of the environment, it is thought that priors are encoded implicitly by the organization of synaptic connections in sensory areas^50,51^. In sensorimotor and cognitive tasks, Bayesian inference is thought to occur later in the association and premotor cortex^23,52^. In the domain of time, there is strong evidence that humans rapidly learn and utilize prior distributions of time intervals to optimize their performance^2,3,10^. In this study, we examined which substrate would be best-suited to acquire prior distributions of time intervals within the sub-second to seconds range, which is also crucial for sensorimotor behaviors.

Two general lines of reasoning led us to hypothesize that the cerebellum may be a key node for Bayesian timing. First, converging evidence from human patients and animal neurophysiology suggests that the cerebellum plays a central role in timing tasks^13,18^. Second, the cerebellum is thought to be involved in acquiring and updating internal models of movement^53,54^, which implies that the cerebellum has the capacity to learn temporal contingencies that relate sensory inputs to motor outputs and vice versa. This led us to the examine whether the cerebellum could support learning distributions of time intervals and support Bayesian timing.

We constructed a model based on the known anatomy and physiology of the cerebellum with three specific components, a basis set, a learning rule, and an integrated output. The basis set assumption is consistent with recent studies indicating that granule cells exhibit temporally heterogeneous activity patterns^24,25^. We modeled the basis set by assuming that GCs form a temporal basis set composed of Gaussian kernels. This is a simplification as each GC is likely to be activated at multiple time points and the detailed temporal structure of the basis set is likely to depend on task-dependent extra-cerebellar inputs^29,55^ from mossy fibers. Nonetheless, the massive convergence of GCs to PCs, coupled with heterogeneous activity patterns in GCs, suggests that the input to each PC can be modeled by a temporally dense basis set. A key assumption in the construction of the basis set was that GC activity is subject to cumulative temporal noise resulting in trial-by-trial jitter in GC activity. What motivated this assumption was the presence of scalar variability in a variety of timing tasks^6^ including those in which the cerebellum is thought to be involved^7,9^. This can be experimentally verified by evaluating whether trial-averaged activity of granule cells attenuates over time and becomes more variable.

The learning rule in TRACE consists of LTD that weakens eligible GC–PC synapses upon the activation of CFs, and LTP, which restores synaptic weights in the absence of CF activity. Although this formulation is relatively standard, complementary plasticity mechanisms might also be at play as recent work has begun to demonstrate^30,32^. For example, plasticity mechanisms in GC–PC synapses may be tuned to diverse intervals and may be region and task dependent^56^ providing complementary substrates for learning time intervals. Similarly, certain aspects of learning are thought to depend on intracellular PC mechanisms^57^. Future work might seek to augment the standard learning rules in TRACE based on the relevance of alternative plasticity mechanisms for learning time interval distributions.

In the context of RSG, we examined the consequence of Ready and Set visual flashes activating GCs and CFs, respectively. There are numerous lines of evidence suggesting that both GCs and CFs can be activated by visual signals. For example, anatomical studies have shown that the lateral geniculate nucleus^58^, the superficial, intermediate, and deep layers of the superior colliculus^59^, and the visual cortex^60^ project to different regions of the pontine nuclei. Moreover, stimulation of these areas in lieu of a conditioned stimulus is sufficient to induce eyeblink conditioning^61^. Therefore, there are numerous anatomical pathways for visual flashes to drive GCs. Visual input may also be relayed to the cerebellar cortex by the inferior olive through visual afferents, such as the superior colliculus^62^. Consistent with this view, visual flashes have been shown to evoke complex spikes in the lateral cerebellum^63^.

The last component of the model is the integration of PC activity in DN. DN may be play an important role in conveying timing information during non-motor tasks^18,64^. For example, recent physiology work suggests an intriguing role for DN in a temporal oddball detection task^18^ which goes beyond traditional motor functions attributed to the cerebellum. A potential non-motor function for DN was also noted by anatomical studies showing that DN interacts with higher cortical areas that do not directly drive movements^45,65^. Of particular relevance to our work is the interaction between DN and the parietal and supplementary motor cortical areas^66^. These areas have been implicated in a range of timing tasks^23,67^, and the activity of parietal cortical neurons in the context of the RSG task matches the predictions of TRACE remarkably well (Fig. 6).

Our assumption that DN neurons integrate PC activity was motivated by the autaptic organization of large columnar neurons in DN^39^ (Fig. 1c). As numerous theoretical studies have shown, this organization is well-equipped to carry out temporal integration^40,41^. Indeed, the match between the output of TRACE and LIP activity in the RSG task (Fig. 6) as well the ability of TRACE to emulate Bayesian inference (Figs. 2, 4) was afforded by the assumption of integration. Our model therefore makes a general prediction that certain DN neurons integrate the signals they receive from both PCs and other extra-cerebellar inputs. Stated differently, TRACE predicts that PC activity carries a time derivative of the cerebellar output provided by DN. As we have shown (Fig. 8a–c), this concept could be extended to different tasks with different cerebellar output circuits, where the dynamics of the behavioral output (e.g., eyelid position) is modulated by the prior distribution. This idea is consistent with observations that PCs carry other signals that may represent the time derivative of behavioral outputs, such as the speed of hand movements^68^, speed of saccades^69^, speed of smooth pursuit^70^, and speed of eyelid in eyeblink conditioning^71^. We note however, that this integration may also occur further downstream in parietal or frontal cortical areas^72,73^, which receive transthalamic input from DN^44,45^ and have been implicated in temporal integration of information^23,72^.

TRACE provides a circuit-level description of how the brain could acquire and utilize prior distributions of time intervals. The underlying computation however, does not rely on explicit representations of the traditional components of Bayesian models including the prior, the likelihood and the posterior. Instead, the output of the model represents an online estimate of elapsed time that derives from a composite representation of the prior and the likelihood within the cerebellar cortex. This is equivalent to directly mapping a measurement of elapsed time to its Bayesian estimate through a nonlinear transformation, as previously hypothesized^3^.

Although, we found the cerebellar circuitry to be particularly suitable for learning prior distributions of sub-second to second time intervals, it is almost certain that the richness of temporal processing depends on coordinated interactions of the cerebellum with other brain areas including cortex, the basal ganglia and hippocampus. For example, in the RSG task, evaluation of behavioral performance is likely to engage higher cortical areas, and the error-driven correction of timing performance is likely to engage the basal ganglia^74^. Furthermore, numerous studies have found neural correlates of interval timing across the cortico-basal ganglia circuits^67,75^. Finally, learning of time intervals in the cerebellum may depend on inputs from other brain areas, especially in cases where continuous sensory drive is not present, such as in trace eyeblink conditioning where persistent activity may be supplied by forebrain regions^29,76^.

In sum, our work highlights the potential for an exciting new function for the cerebellum; the ability to represent prior distributions of time intervals. Remarkably, this function emerges naturally from what is known about the anatomy and physiology of the cerebellum in the context of tasks that require exposure to a range of time intervals. The simplicity and success of TRACE as a model for Bayesian behavior and its underlying neural signatures invites a serious consideration of the possibility that the cerebellum is a key component of circuits that the brain uses to emulate probabilistic computations in the domain of time.

## Methods

### Bayesian estimator

The RSG time interval estimation task consists of two consecutive cues, Ready and Set, that mark an interval (*t*_s_) drawn from a prior distribution *π*(*t*_s_). Participants measure *t*_s_ and subsequently reproduce it (Fig. 1a). Following previous work^3^, we modeled the measured interval, *t*_m_ as drawn from a Gaussian distribution centered at *t*_s_ whose standard deviation is proportional to *t*_s_ with coefficient of variation *w*_m_.

$$p\left( {t_{\rm m}|t_{\rm s}} \right) = \frac{1}{{\sqrt {2\pi \left( {w_{\rm m}t_{\rm s}} \right)^2} }}{\rm e}^{\frac{{ - \left( {t_{\rm s} - t_{\rm m}} \right)^2}}{{2\left( {w_{\rm m}t_{\rm s}} \right)^2}}}$$

The Bayes-Least-Square (BLS) estimate *t*_e_ is the expected value of the posterior distribution *p*(*t*_s_|*t*_m_), as follows:

$$p\left( {t_{\rm s}|t_{\rm m}} \right) = \frac{{\pi \left( {t_{\rm s}} \right)p\left( {t_{\rm m}|t_{\rm s}} \right)}}{{{\int} {\pi \left( {t_{\rm s}} \right)p\left( {t_{\rm m}|t_{\rm s}} \right)} }}$$

$$t_{\rm e} = f\left( {t_{\rm m}} \right) = E\left[ {p\left( {t_{\rm s}|t_{\rm m}} \right)} \right]$$

where *E*[.] denotes expectation. The BLS estimator can be formulated as a deterministic nonlinear function, *f*(*t*_m_), that maps a noisy *t*_m_ to an optimal estimate *t*_e_ (Fig. 1b). In the manuscript, we assessed the behavior of the BLS estimator for different uniform and Gaussian priors and for *w*_m_ = 0.1. This value is consistent with previous reports for humans performing the RSG task^3^. In Fig. 2, predictions of all models were generated for 1000 samples *t*_s_ (uniformly drawn from 600 to 1200 ms), a Weber fraction of 0.1 was used to generate 10,000 *t*_m_ values for each of these. A distribution of root-mean squared errors (RMSE) was computed in 1000 repeated runs of this process.

### TRACE model

In TRACE, Purkinje cells (PCs) receive input from *N* granule cells (GCs). The spike count (*y*) for the *i*th GC follows an inhomogeneous Poisson process with a Gaussian rate function *ω*(*t*) centered at *μ*_*i*_ with standard deviation *σ*_*i*_. The time of maximum firing rate for the *i*th GC, *μ*_*i*_, is specified with respect to the time of Ready.

$$\begin{array}{*{20}{l}} {p\left( {y_i|t} \right)} \hfill &  = \hfill & {\frac{1}{{y_i!}}\omega\left( t \right)^{y_i}{\rm e}^{ - \omega \left( t \right)}} \hfill \\ {\omega \left( t \right)} \hfill &  = \hfill & {\frac{1}{{\sqrt {2\pi \sigma _i^2} }}{\rm e}^{ - \frac{{\left( {t - \mu _i} \right)^2}}{{2\sigma _i^2}}}} \hfill \end{array}$$

Due to scalar variability, the internal estimate of elapsed time $\left( {\tilde t} \right)$ has a probabilistic relationship to the objective elapsed time (*t*). We formulated this relationship as a conditional Gaussian probability distribution whose mean is (*t*), and standard deviation scales with (*t*) by a scaling factor *w*_b_. This scaling factor is analogous to the Weber fraction introduced for the behavioral modeling. To model the basis set in the presence of this variability, we derived the expectation of the basis set across trials as a function of elapsed time, *t*, by marginalizing over this distribution.

$$\begin{array}{*{20}{l}} {p\left( {\tilde t|t} \right)} \hfill &  = \hfill & {\frac{1}{{\sqrt {2\pi \left( {w_{\rm b}t} \right)^2} }}{\rm e}^{\frac{{ - \left( {\tilde t - t} \right)^2}}{{2\left( {w_{\rm b}t} \right)^2}}}} \hfill \\ {p\left( {y_i|t} \right)} \hfill &  = \hfill & {\mathop {\int}\limits_{\tilde t} {p\left( {y_i|\tilde t} \right)p\left( {\tilde t|t} \right){\rm d}\tilde t} } \hfill \\ {p\left( {y_i|t} \right)} \hfill &  = \hfill & {\frac{1}{{y_i!}}\frac{1}{{\sqrt {2\pi \left( {w_{\rm b}t} \right)^2} }}{\kern 1pt} \mathop {\int}\limits_{\tilde t} {\omega \left( {\tilde t} \right)^{y_i}{\rm e}^{ - \omega \left( {\tilde t} \right)}{\rm e}^{\frac{{ - \left( {\tilde t - t} \right)^2}}{{2\left( {w_{\rm b}t} \right)^2}}}} } \hfill \end{array}$$

This transformation results in two forms of inhomogeneity across the basis set kernels: (1) it reduces the amplitude of kernels as a function of time, and (2) it causes kernels to become wider as a function of time (Supplementary Fig. 1). As expected, inferring elapsed time from this perturbed basis set using a maximum likelihood decoder produces scalar variability (Supplementary Fig. 1).

We introduced a simplified parameterization to capture these two inhomogeneities. The reduction in amplitude was modeled by a decaying exponential function, *g*(*t*), with time constant *τ*_basis_, and the increase in width was modeled as a linear function, $\sigma _{{\rm basis}_i} = \sigma _{\rm o}i\kappa {\mathrm{/}}N$, where *i* indexes neurons ordered according to their preferred time interval, *N* is the total number of neurons (*N* = 500) and *κ* is the proportion of increase in the width *σ*_o_. A detailed analysis of the robustness of model predictions upon varying these parameters can be found in Supplementary Fig. 5. The resulting function that describes the rate of the *i*th GC is:

$$r_i\left( t \right) = g\left( t \right){\textstyle{1 \over {\sqrt {2\pi \sigma _{{\rm basis}_i}^2} }}}{\rm e}^{\frac{{ - \left( {t - t_i} \right)^2}}{{2\sigma _{{\rm basis}_i}^2}}}$$

where $$g(t) = {\rm e}^{\frac{{ - t}}{{\tau _{{\rm basis}_i}}}}$$.

In the model, the PC activity is computed as a weighted sum of GC activity.

$$V_{{\rm pc}} = {\sum} {r_i(t)w_i}$$

where *w*_*i*_ represents the synaptic weight of the *i*th GC and $V_{{\rm pc}}^\prime = - V_{{\rm pc}}$.

Similar to previous work^27^, LTD in TRACE is modeled for each GC–PC synapse as proportional to the rate of firing of respective GCs shortly before the firing of climbing fibers (CFs) at the time of Set. The time before CF firing at which GC–PC synapses become eligible for LTD is called the eligibility trace (*ε*), which we assume occurs 50 ms before Set in the model. We assume that the CF fires instantaneously at the time of Set and is zero at all other times. In the absence of CF stimulation and in the presence of GC firing, a weak restoring force (LTP) acts to reverse learning. The dynamics of LTD and LTP was governed by their respective time constants, *τ*_ltd_ and *τ*_ltp_. A more detailed analysis of variation of these parameters can be found in Supplementary Fig. 3. In the absence of learning, synapses would gradually drift toward the baseline, *w*_o_.

$$\frac{{{\rm d}w_i}}{{{\rm d}t}} = - \frac{1}{{\tau _{{\rm ltd}}}}r_i\left( {t_{\rm s} - \varepsilon } \right)\delta(t-t_{\rm s}) + \frac{1}{{\tau _{{\rm ltp}}}}\left( {w_{\rm o} - w_i} \right)$$

The presence of the eligibility trace implies that any CF firing at the onset of Ready will have no bearing upon the plasticity of the GC–PC synapses. Similarly, GC activation at the time of Set will be irrelevant to learning of the prior. Further, our results remain qualitatively unchanged under assumptions of more complex functions of the eligibility trace.

Purkinje cells (PCs), which constitute the sole output of the cerebellar cortex are inhibitory. Since LTD reduces PC output, LTD-dependent learning has a net excitatory effect on the membrane potential of dentate neurons *V*_dn_. Furthermore, individual neurons in the dentate nucleus (DN) receive distal synaptic input from both extra-cerebellar afferents, mossy and climbing fibers. We assume this effective input to be constant (*I*_eff_). Finally, large columnar DN neurons are rich in axon collaterals that make autaptic connections^39^. With these elements, the membrane potential of DN neurons can be modeled as:

$$\frac{{{\rm d}V_{{\rm dn}}\left( t \right)}}{{{\rm d}t}} = - V_{{\rm dn}}\left( t \right) + g_{{\rm dn}}V_{{\rm dn}}\left( t \right) - g_{{\rm pc}}V_{{\rm pc}}\left( t \right) + I_{{\rm eff}}$$

where *g*_dn_ and *g*_pc_ correspond to conductances associated with the autaptic input and PCs, respectively. For the simulations in the main text, we set *g*_dn_ to 1, which corresponds to perfect integration, and set *I*_eff_ to a fixed value equal to the average PC activity so that DN neurons receive similar levels of excitation and inhibition. However, TRACE exhibits robust Bayesian behavior under significant variation of both *I*_eff_ and *g*_dn_ (Supplementary Fig. 4). With these assumptions, the model can be simplified as follows:

$$V_{{\rm dn}}(t) = {\int} {\left( {I_{{\rm eff}} - g_{{\rm pc}}V_{{\rm pc}}\left( t \right)} \right){\rm d}t}$$

We simulated trial-by-trial dynamics by generating a spiking model for TRACE. On each trial and for each GC, we generated spike-trains according to a non-homogenous Poisson process whose rate was specified by the corresponding kernel in the basis set. Spikes were convolved with an excitatory postsynaptic gaussian kernel with standard deviation 20 ms. GC–PC synapses underwent LTD according to the level of activity of corresponding GCs at the time *ε* (eligibility trace) before the time of Set.

### Parameter values

The steady state behavior of TRACE is primarily governed by the parameters of the basis set and its dynamics by the learning rate parameters. The basis set has three parameters; *τ*_basis_, specifying attenuation, and *σ*_o_, *κ*, specifying increase in width. In Figs. 1–6, the basis set parameters were *σ*_o_ = 100 ms, *κ* = 0.2 and *τ*_basis_ ranges from 500 to 1000 ms. The TRACE model can tolerate a wide range of variation in these parameters (Supplementary Fig. 5).

The learning dynamics in TRACE are characterized by changes in the magnitude of synaptic weights (*A*_eff_) and an effective time constant (*τ*_eff_) needed to reach such magnitudes. These variables are controlled by the *τ*_ltd_ and *τ*_ltp_ and the width of the prior distribution. In Supplementary Fig. 3, we show how *A*_eff_ and *τ*_eff_ vary with *τ*_ltd_ and *τ*_ltp_. In Fig. 5b, we used the prior distributions used in a previous study^2^ and report the average deviation (across 100 simulations) of the PC activity in bins of 20 trials with *τ*_ltd_ = 100 and *τ*_ltp_ = 300 ms. Synaptic weights were not allowed to fall below zero and the lowest permissible value for long-term depression was *τ*_ltd_ = 50 ms.

In Fig. 7a, which shows model behavior with all components intact, we use the same parameters as those used for Figs. 1–6. In Fig. 7b, c, we vary the basis set parameters. There is no exponential decay in Fig. 7b and no widening of sigma. Original kernel width remains *σ*_o_ = 100 ms. In Fig. 7c, we set *τ*_basis_ = 400 ms. All other parameters remain unchanged. In Fig. 7d, we remove the learning equation from the model and do not make any adjustment to *V*_dn_. In Fig. 7e, we remove the integration component of the model.

### Eyeblink conditioning

Human participants (*n* = 10, 5 female, 5 male average age 27.7 ± 2.3) gave informed consent to participate in a trace eyeblink conditioning experiment, which conformed to protocols approved by the medical ethical review committee (Medisch Ethische Toetsingscommissie) at the Erasmus Medical Center, Rotterdam. Participants viewed a documentary video while they received repeated pairings of conditioned and unconditioned stimuli (CS and US) every 5–10 s (random inter-trial-interval). The CS was a 50 ms auditory tone (Gaussian envelope with sd 25 ms, 1 kHz). The US was a 50-ms peri-occular airpuff (40 psi) and was delivered via a lightweight headset equipped with an integrated tube that administered a precisely timed airpuff at a minimum distance of 3 cm from the left eye without obstructing the participant’s view. Eye movements were captured by a high-speed camera (333-frame/s, scA640-120 gc, Basler) integrated with a custom data acquisition system Blink.

During the first 144 trials, the inter-stimulus-interval (ISI) for CS–US paired trials was drawn from a uniform distribution with five values ranging from 529 to 1029 ms (wide prior). Thereafter, unbeknownst to the participants, the ISI switched to either a fixed 1029 ms or a fixed 529 ms interval (narrow/delta prior) and continued for another 48 trials. An equal number of participants were randomly assigned to each condition (529 or 1029). Throughout the session, we included randomly interspersed CS-only “test trials” (i.e., no US), of which there were 24 during the first 144 trials before the switch, and 12 during the 48 trials after the switch. We measured the distributions of baseline eyelid positions prior to CS, and eyelid closure responses that exceeded 3 standard deviations from the baseline were considered to be conditioned responses (CR). CR traces were smoothed using a Gaussian kernel with SD = 30 ms and normalized to the full blink range. The full blink range was computed as the difference between minimal closure measured from average baseline and full closure measured from the peak of the unconditioned response. The earliest point at which maximum closure was reached within a trial, was taken as the maximum closure time. We denoted the difference between maximum closure time and the mean of the wide prior distribution as ‘deviation’ (Fig. 8c).

### Synchronization continuation

Seven human participants (2 female, 5 male) gave informed consent to perform the study, which conformed to protocols approved by the COUHES (Committee on the Use of Humans as Experimental Subjects) at the Massachusetts Institute of Technology. Each participant completed one training session and one test session each lasting 50 min. Data from one participant were not included in analyzes due to a large change in behavior during the test session. Participants completed between 241 and 350 trials in the test session. The analyzes were performed on the test session only. Participants viewed all stimuli binocularly from a distance of approximately 67 cm on either a 23-inch Apple A1082 LCD monitor at a resolution of 1900 × 1200 driven by an Intel Macintosh Mac Pro computer, or a 24-inch early 2009 Apple Mac Pro at a refresh rate of 60 Hz in a dark, quiet room. Responses were registered on a standard Apple Keyboard connected to the experimental machine. Each trial began with the presentation of a red circular fixation stimulus (diameter = 0.75 deg visual angle) in the center of the screen. After a variable delay (200 ms plus an additional amount of time which was exponentially distributed with mean = 300 ms and a maximum value of 2300 ms), a synchronization stimulus was flashed four times with its pacing interval (PI) chosen from a discrete uniform distribution (five intervals, minimum = 550 ms, maximum = 817 ms). The flashing stimulus was a gray annulus centered around fixation; ID = 1 deg, OD = 1.25 deg. Participants were instructed to tap a response key synchronously with the third and fourth synchronization flashes and continue tapping to reflect the same pacing interval until instructed to stop. The number of continuation taps required was three plus an exponentially distributed number with mean of nine and maximum of 22. The first inter-tap interval was defined as the interval between the middle of the second flash and the first key press. Subsequent inter-tap-intervals (ITIs) were defined as the interval between successive key presses. If a participant’s ITI became greater than or less than the 50% of the target interval, the trial was immediately aborted. No other feedback was given.

In the synchronization-continuation task, we fit participants’ responses to a Bayesian observer model previously shown to capture the behavior of human subjects in the RSG task^3^. Briefly, the model consists of three stages: a noisy measurement stage, the BLS Bayesian estimator previously described (see “Bayesian estimator”), and a noisy production stage. Noise in the measurement and production phases were formulated as zero-mean Gaussian with standard deviation scaling with the base interval (see previous work^3^ for details). Bias towards the mean of the prior was characterized with the statistic Bias, which summarizes the difference between average and correct ITI responses and is defined as

$${\rm Bias} = \sqrt {\mathop {\sum}\limits_{i = 1}^N {{\rm bias}_i^2} }$$

where bias_*i*_ is the difference between the mean ITI response and correct response for pacing interval *i* of the *N* intervals constituting the prior (*N* = 5).

### Data availability

The data that support the findings of this study are available from the corresponding author upon reasonable request.

## Electronic supplementary material

Supplementary Information
