# Supplementary material for: A cerebellar mechanism for learning prior distributions of time intervals
Source: Nat Commun. 2018 Feb 1;9:469. doi: 10.1038/s41467-017-02516-x (PMC5794805; doi:10.1038/s41467-017-02516-x)
Supplement: Supplementary file 1 — Supplementary Information [file 41467_2017_2516_MOESM1_ESM.pdf]

Supplementary Information

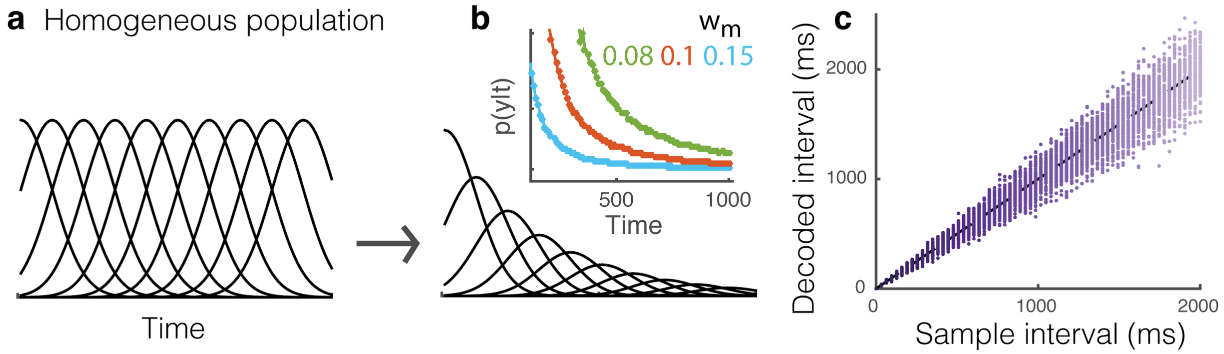

Supplementary Figure 1: Probabilistic motivation of the basis set. a) A homogeneous basis set in the absence of scalar noise (sub-sampled). b) The expected profile of the same basis set under the influence of scalar noise. This causes the expected basis set to attenuate and become wider with time. The inset shows the profile of attenuation for three weber fractions (w_m). c) A maximum likelihood estimate of time decoded from the activity of the modified basis set expresses scalar variability.

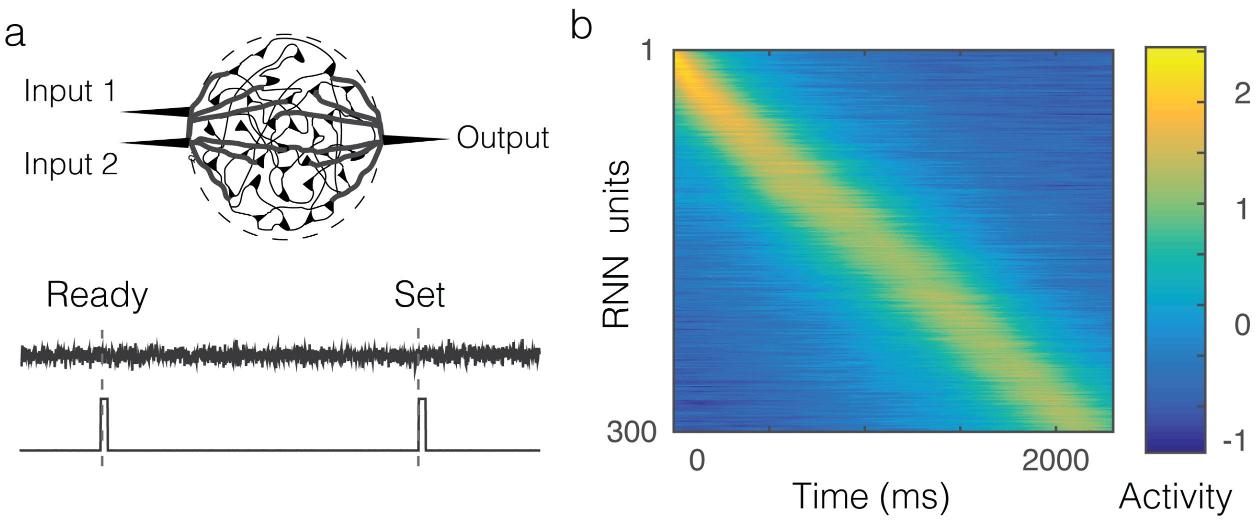

Supplementary Figure 2. Demonstration of the effect of scalar variability on the basis set in a random recurrent network. a) a recurrent neural network schematic (top). Network is driven by white noise and transient pulses associated with Ready and Set (below). b) Trial-averaged activity of all the units rank-ordered by peak activity as a function of time.

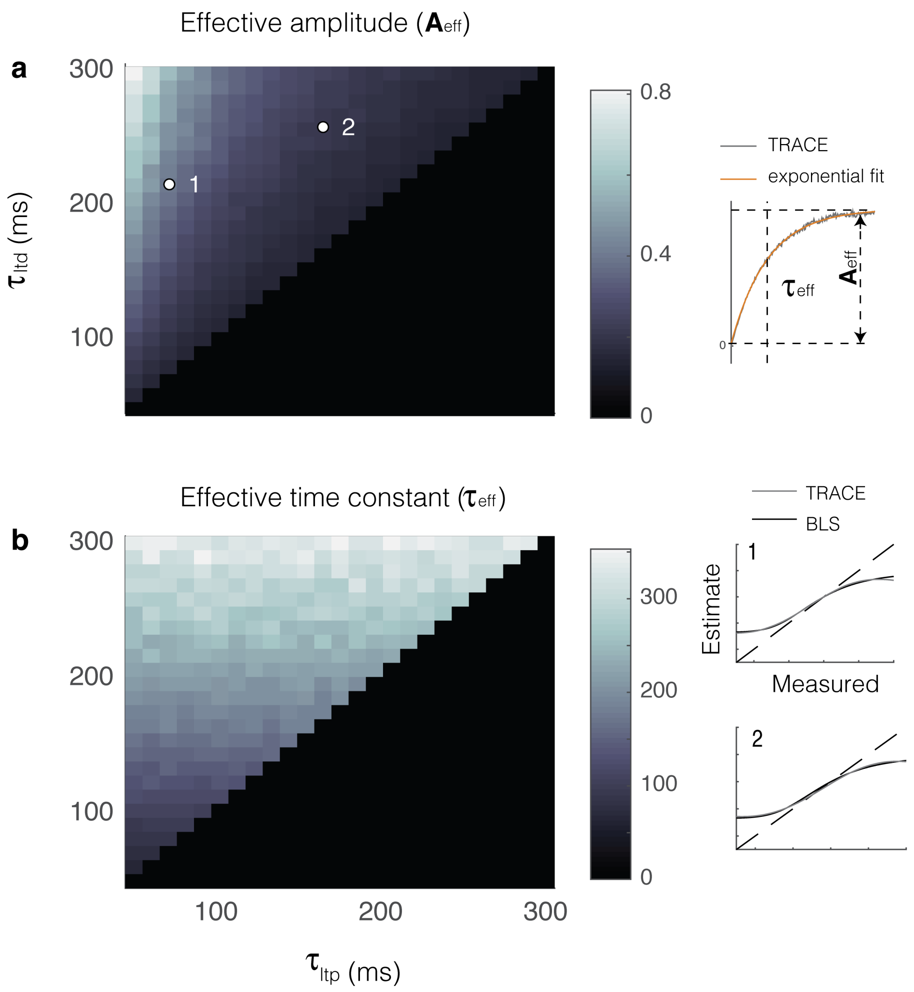

Supplementary Figure 3. Learning dynamics. a) Grayscale indicates the effective amplitude (A_{eff}) of synaptic weights across all neurons as a function of the time constants of LTD and LTP (τ_{ltd} and τ_{ltp}). The inset shows how A_{eff} and τ_{eff} were determined from sum of synaptic weights across neurons over 200 observations. Inset below shows linearly transformed integrated TRACE outputs (gray) compared to a Bayes least-squares function (black) for two arbitrary points marked in a). b) Grayscale indicates effective time constant τ_{eff} as a function of τ_{ltd} and τ_{ltp}.

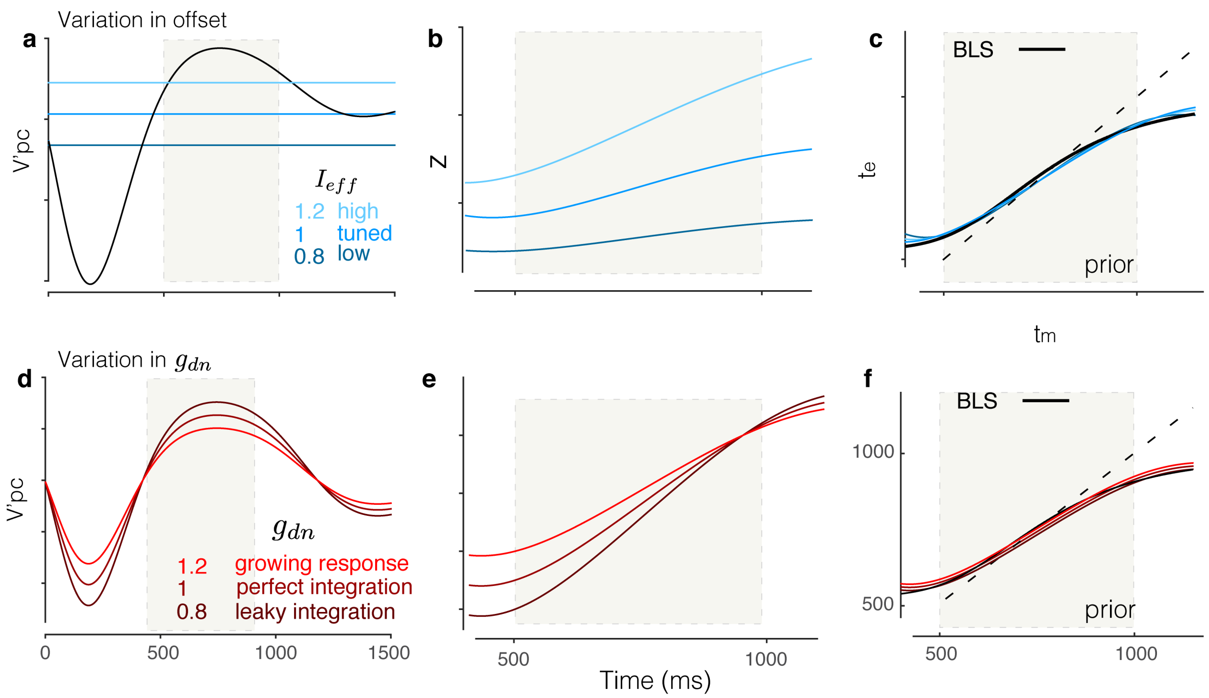

Supplementary Figure 4. Variation of the parameters of integration in TRACE. a-c) The effect of varying I_{eff} (shades of blue) on model behavior. a) V'_{pc} as a function of time (black) with the three offset levels overlaid. b) The integrated output with the three offsets. c) Linearly transformed output for the three offsets. The black shows the Bayes least-squares function; i.e., Bayesian interval estimate (t_e) as a function of the measured interval (t_m). The dark to light blue correspond to cases when the offset is 20% lower (low), equal (tuned), or 20% higher (high) than the mean of V'_{pc}. d-f) The effect of varying g_{dn} (shades of red) on model behavior. Panels follow the same organization as the top row. The dark to light red correspond to a leaky integration (g_{dn} = 0.8), perfect integration (g_{dn} = 1), and a case where responses grow faster than perfect integration (g_{dn} = 1.2). If g_{dn} is significantly greater than 1, the neuron becomes unstable and the model breaks down. The shaded area in all panels correspond to the support of the prior distribution.

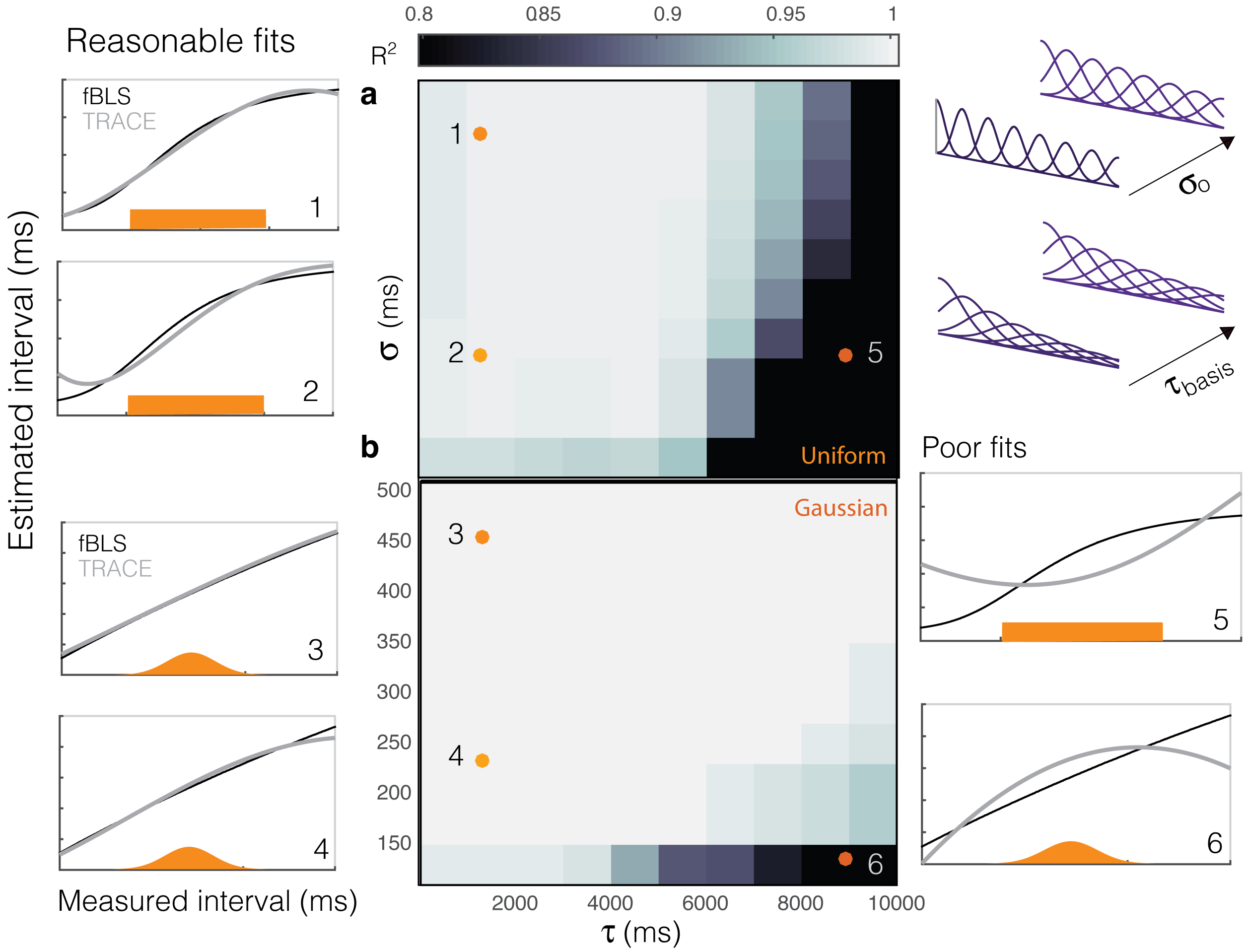

Supplementary Figure 5. Robustness to model parameters. A grayscale heatmap showing the goodness of fit between the TRACE model (gray line) and Bayes least-squares (BLS - black line) while varying parameters of the basis set (kernel width σ_o and time constant of exponential decay τ_{basis}) for a) uniform and b) Gaussian prior distributions (orange). Plots on the left (1-4) and right (5-6) show examples with high and low R^2, respectively.
